# Lithium Therapy Improves Neurological Function and Hippocampal Dendritic Arborization in a Spinocerebellar Ataxia Type 1 Mouse Model

**DOI:** 10.1371/journal.pmed.0040182

**Published:** 2007-05-29

**Authors:** Kei Watase, Jennifer R Gatchel, Yaling Sun, Effat Emamian, Richard Atkinson, Ronald Richman, Hidehiro Mizusawa, Harry T Orr, Chad Shaw, Huda Y Zoghbi

**Affiliations:** 1 21st Century COE program on Brain Integration and Its Disorders, Tokyo Medical and Dental University, Tokyo, Japan; 2 Department of Molecular and Human Genetics, Baylor College of Medicine, Houston, Texas, United States of America; 3 Howard Hughes Medical Institute, Baylor College of Medicine, Houston, Texas, United States of America; 4 Department of Neuroscience, Baylor College of Medicine, Houston, Texas, United States of America; 5 Department of Pediatrics, Baylor College of Medicine, Houston, Texas, United States of America; 6 Institute of Human Genetics, University of Minnesota, Minneapolis, Minnesota, United States of America; Harvard University, United States of America

## Abstract

**Background:**

Spinocerebellar ataxia type 1 (SCA1) is a dominantly inherited neurodegenerative disorder characterized by progressive motor and cognitive dysfunction. Caused by an expanded polyglutamine tract in ataxin 1 (ATXN1), SCA1 pathogenesis involves a multifactorial process that likely begins with misfolding of ATXN1, which has functional consequences on its interactions, leading to transcriptional dysregulation. Because lithium has been shown to exert neuroprotective effects in a variety of conditions, possibly by affecting gene expression, we tested the efficacy of lithium treatment in a knock-in mouse model of SCA1 (*Sca1^154Q/2Q^* mice) that replicates many features of the human disease.

**Methods and Findings:**

*Sca1^154Q/2Q^* mice and their wild-type littermates were fed either regular chow or chow that contained 0.2% lithium carbonate. Dietary lithium carbonate supplementation resulted in improvement of motor coordination, learning, and memory in *Sca1^154Q/2Q^* mice. Importantly, motor improvement was seen when treatment was initiated both presymptomatically and after symptom onset. Neuropathologically, lithium treatment attenuated the reduction of dendritic branching in mutant hippocampal pyramidal neurons. We also report that lithium treatment restored the levels of *isoprenylcysteine carboxyl methyltransferase* (*Icmt;* alternatively, *Pccmt),* down-regulation of which is an early marker of mutant ATXN1 toxicity.

**Conclusions:**

The effect of lithium on a marker altered early in the course of SCA1 pathogenesis, coupled with its positive effect on multiple behavioral measures and hippocampal neuropathology in an authentic disease model, make it an excellent candidate treatment for human SCA1 patients.

## Abstract 2

Huda Zoghbi and colleagues show that lithium treatment initiated before or after disease onset improves multiple symptoms of neurodegeneration in a mouse model of spinocerebellar ataxia.

## Editors' Summary

**Background.:**

Spinocerebellar ataxia type 1 (SCA1) is an inherited, incurable neurodegenerative disease in which the neurons (cells that transmit information between the brain and body) in the cerebellum (the brain region that coordinates movement) gradually die. Symptoms of the disease, which usually begins in early adult life, include poor coordination of movement (ataxia), slurred speech, and cognitive (learning and memory) problems. As more neurons die, these symptoms get worse until breathing difficulties eventually cause death. SCA1 is a “triplet repeat disease.” Information for making proteins is stored in DNA as groups of three nucleotides (codons), each specifying a different amino acid (the building blocks of proteins). In triplet repeat diseases, patients inherit a mutant gene containing abnormally long stretches of repeated codons. In SCA1, the repeated codon is CAG, which specifies glutamine. Consequently, SCA1 is a “polyglutamine disease,” a group of neurodegenerative disorders in which an abnormal protein (a different one for each disease) containing a long stretch of glutamine forms nuclear inclusions (insoluble lumps of protein) in neurons that, possibly by trapping essential proteins, cause neuronal death. In SCA1, the abnormal protein is ataxin 1, which is made in many neurons including the cerebellar neurons (Purkinje cells) that coordinate movement.

**Why Was This Study Done?:**

Early in SCA1, the production of several messenger RNAs (the templates for protein production) decreases, possibly because transcription factors (proteins that control gene expression) interact with the mutant protein. Could the progression of SCA1 be slowed, therefore, by using an agent that affects gene expression? In this study, the researchers have investigated whether lithium can slow disease progression in an animal model of SCA1. They chose lithium for their study because this drug (best known for stabilizing mood in people with bipolar [manic] depression) affects gene expression, is neuroprotective, and has beneficial effects in animal models of Huntington disease, another polyglutamine disease.

**What Did the Researchers Do and Find?:**

The researchers bred mice carrying one mutant gene for ataxin 1 containing a very long CAG repeat and one normal gene (*Sca1^154Q/2Q^* mice; genes come in pairs). These mice develop symptoms similar to those seen in people with SCA1. After weaning, the mice and their normal littermates were fed normal food or food supplemented with lithium for several weeks before assessing their ability to coordinate their movements and testing their cognitive skills. Dietary lithium (given before or after symptoms appeared) improved both coordination and learning and memory in the *Sca1^154Q/2Q^* mice but had little effect in the normal mice. Lithium did not change the overall appearance of the cerebellum in the *Sca1^154Q/2Q^* mice nor reduce the occurrence of nuclear inclusions, but it did partly reverse hippocampal neuron degeneration in these animals. The researchers discovered this effect by examining the shape of the hippocampal neurons in detail. These neurons normally have extensive dendrites—branch-like projections that make contact with other cells—but these gradually disappear in *Sca1^154Q/2Q^* mice; lithium partly reversed this loss. Finally, lithium also restored the level of *Icmt/Pccmt* mRNA in the cerebellum to near normal in the *Sca1^154Q/2Q^* mice—this mRNA is one of the first to be reduced by ataxin 1 toxicity.

**What Do These Findings Mean?:**

These findings show that treatment with lithium slows neurodegeneration in a mouse model of SCA1, even when it is given only after the first symptoms of the disease have appeared. Unfortunately, lithium did not improve the life span of the *Sca1^154Q/2Q^* mice (although this could be because the mutant SCA1 protein has some deleterious effects outside the brain). Thus, lithium is unlikely to cure SCA1, but it could provide some help to people with this devastating disease, even if (as is usual), their condition is not diagnosed until the disease is quite advanced. However, because drugs that work in animal models of diseases do not necessarily work in people, patients with SCA1 (or other polyglutamine diseases, which might also benefit from lithium supplementation) should not be treated with lithium until human trials of this approach have been completed.

**Additional Information.:**

Please access these Web sites via the online version of this summary at http://dx.doi.org/10.1371/journal.pmed.0040182.

- The US National Ataxia Foundation provides information for patients
- International Network of Ataxia Friends has information for patients and carergivers on ataxias, including SCA1
- GeneTests provides information for health care providers and researchers about SCA1
- Online Mendelian Inheritance in Man (OMIM) has detailed scientific information on SCA1
- Huntington's Outreach Project for Education offers information for lay people from Stanford University on trinucleotide repeat disorders including SCA1

## Introduction

Spinocerebellar ataxia type 1 (SCA1) is one of nine known dominantly inherited neurodegenerative disorders caused by the expansion of a CAG repeat that encodes polyglutamine in the respective disease protein [1]. Patients with SCA1 suffer from progressive loss of motor coordination and some cognitive deficits. Pathologically, SCA1 is characterized by degeneration of Purkinje cells and brain stem neurons, but eventually generalized atrophy sets in [2–4].

There is strong evidence that expanded polyglutamine tracts cause the host protein to misfold [5], but polyglutamine pathogenesis is a complex, multifactorial process. In SCA1, the nuclear localization of the mutant protein [6], protein context, and protein modification are all factors in disease expression [7,8]. Emamian et al. [7] demonstrated that serine 776 (S776) in ataxin 1 (ATXN1) is essential for its pathogenicity in transgenic mice; Chen et al. [8] showed that phosphorylation of this residue by Akt enables 14-3-3 to interact with and stabilize ATXN1. Genetic studies revealed that overexpression of Hsp70 suppresses ATXN1-induced degeneration, possibly through its effect on the misfolded protein [9]. We sought to determine whether we could improve the neurological dysfunction of SCA1 mutant mice by using pharmacologic therapy that targets one of the potential early downstream consequences of misfolded ATXN1. An early event in the pathogenic cascade is the down-regulation of the mRNAs of several genes. Lin et al. demonstrated dysregulation of several neuronal genes in the Purkinje cells of SCA1 transgenic mice even before the onset of behavioral or neuropathological changes, and they observed similar changes in the tissue of humans with SCA1 [10]. Therefore, we rationalized that if we use a drug that enhances gene expression, we might slow SCA1 progression. Lithium was recently shown to exert neuroprotective effects, possibly by affecting gene transcription [11,12]. Lithium is widely known for its use in the treatment of bipolar disease. The mechanism of lithium's mood-stabilizing activity has not been elucidated, in part because lithium exerts its effects on a wide range of cellular functions: for example, it inhibits inositol production, affects the protein kinase C signaling pathway, and inhibits glycogen synthase kinase 3 (GSK3) [13–15]. Furthermore, several studies in cellular and animal models have shown that lithium can suppress neurodegeneration induced by various kinds of insults, such as Alzheimer disease amyloid-β peptide [16], glutamate [17], and ischemia [17]. Carmichael et al. reported that lithium treatment partially rescued cell death in cells overexpressing Huntington disease exon 1 fragment with 74 glutamines, possibly by inhibiting GSK3 and subsequently altering gene transcription [11].

The *Sca1^154Q/2Q^* knock-in mouse model bears 154 CAG repeats in the mouse *Sca1* locus and expresses full-length mutant ATXN1 in its endogenous expression pattern and context [18]. *Sca1^154Q/2Q^* mice reproduce many features of the human disease, including progressive motor dysfunction and cognitive impairment [18], and therefore allow us to examine a range of effects of lithium treatment on SCA1 pathogenesis in vivo.

## Methods

### Mice and Treatment Regimen

*Sca1^154Q/2Q^* mice and their wild-type littermates were obtained from crossings between male *Sca1^154Q/2Q^* mice and wild-type female mice on two different backgrounds: mice of C57BL/6J–129/SvEv mixed background; and mice that were obtained after C57BL/6J–129/SvEv mutant mice were backcrossed to C57BL/6J at least five times (>N5 generation). They were randomly assigned to two groups either at the time of weaning (presymptomatic) or at five weeks (symptomatic), at which time treatment was initiated. Each group was fed either 0.2% lithium carbonate-containing chow or control Purina rodent chow (Harlan Teklad, http://www.teklad.com). All behavioral tests were conducted by individuals blind to genotype and treatment group. Animals were separated by treatment and three to five animals were housed per cage. Mouse experiments followed protocols approved by the Baylor College of Medicine Institutional Animal Care and Use Committee (IAUAC) and the Institutional Animal Care and Use Committee of Tokyo Medical and Dental University.

### Determination of Serum Lithium Levels

The LI Flex reagent cartridge (Dade Behring, http://www.dadebehring.com) was used on the Dimension clinical chemistry system as an in vitro test to quantitatively measure lithium levels in rodent serum. The LI method of measurement employs a patented compound (lithium dye) that reacts with lithium ions in an alkaline mixture of water and DMSO to form a noncovalent binary complex, the absorbance of which is measured at 540 nm and is directly proportional to lithium concentration in the sample. The LI method of measurement was carried out by the standard operating procedures of the Department of Pathology, Harris County Hospital District, Houston, Texas, United States.

### Rotarod Test

An accelerated rotating rod test (type 7650; Ugo Basile, http://www.ugobasile.com) allowed us to evaluate coordination and motor skill acquisition. Naïve animals at 10 and 13 wk of age were placed on the rod (diameter 3 cm, length 30 cm) in four trials every day for a period of 4 d. Each trial lasted a maximum of 10 min, during which time the rotating rod underwent a linear acceleration from 4 to 40 rpm over the first 5 min of the trial and then remained at maximum speed for the remaining 5 min. The time mice spent on the rod without falling was recorded.

### Morris Maze

Mice aged 9–11 wk were trained in the Morris water maze to locate a hidden platform as described elsewhere [18]. Each mouse was given four trials per day for five consecutive days. After trial 20, each animal was given a probe trial. During the probe trial, the platform was removed and each animal was allowed to search the pool for 60 s.

### Pavlovian Conditioned Fear

Mice were 11–13 weeks of age at the start of testing. Performance in a conditioned fear paradigm was measured as described before [14] using a Freeze Monitor system (San Diego Instruments, http://sandiegoinstruments.com). The test chamber was made of clear Plexiglas and surrounded by a photo beam detection system. The floor of the test chamber was a grid used to deliver an electric shock. A mouse was placed in a test chamber and allowed to explore freely for 2 min. An 80 dB white noise that served as the conditioned stimulus (CS) was then presented for 30 sec, and then followed by a mild foot shock (the unconditioned stimulus, US). Two minutes later, another CS–US pairing was presented. The mouse was removed from the chamber 15–30 s later and returned to its home cage. After 24 h, the mouse was placed back into the test chamber for 5 min and “freezing” behavior was assessed for 5 min (context test). Environmental and contextual cues were changed for the auditory CS test: a white Plexiglas triangular insert was placed in a chamber to alter its shape and spatial cues, the wire grid floor was covered with white Plexiglas, and vanilla extract was placed in the chamber to alter the smell. There were two phases during the auditory CS test. In the first phase (pre-CS), freezing was recorded for 3 min without the CS. In the second phase, the auditory CS was presented, and freezing was recorded for another 3 min. The time mice froze was converted to a percentage freezing value. For the auditory CS test, the percentage freezing value obtained during the pre-CS period was subtracted from the percentage freezing value when the auditory CS was present.

### Open-Field Test

At 2 wk after the conditioned fear test, the *Sca1^154Q/2Q^* mice were placed in the center of an open-field space (40 × 40 × 30 cm). Activity was quantified by a computer-operated Digiscan optical animal activity system (RXYZCM, Acuscan, http://acuscan.com). Each test session was 30 min long, and data were collected in 10 min intervals.

### Immunohistochemistry and Immunofluorescence

Immunohistochemistry and immunofluorescence staining were performed as described elsewhere [18]. Rabbit polyclonal anti-ataxin-1 (11NQ) and monoclonal anti-calbindin antibodies (Sigma, http://www.sigmaaldrich.com) were used for the staining of neuronal intranuclear inclusions (NI) and the dendritic arbors of Purkinje cells, respectively. The quantification of the dendritic arborization was carried out using the NIH Image J software as previously described [18].

### Golgi Staining and Sholl Analysis

*Sca1^154Q/2Q^* mice and their wild-type littermate pairs, between the ages of 24 and 30 weeks, fed on either control or lithium diet from the time of weaning, were used for these experiments (three to six mice per treatment group). Individuals performing experimental manipulations and subsequent analyses were blind to both genotype and treatment group. Golgi staining was carried out using the FD NeuroTechnologies Rapid Golgi Staining Kit according to the manufacturer's protocol (FD NeuroTechnologies, http://www.fdneurotech.com). Sagittal brain sections (60 μm) were prepared and camera lucida drawings were used to trace the morphology of hippocampal CA3 pyramidal neurons. Representative pyramidal cells were drawn at a magnification of 400×. Tracings of 6–10 neurons were made per animal, for a total of 30–60 neurons per treatment group. Sholl analysis of the apical and basilar dendrites of these neurons was then carried out. Briefly, a series of increasingly large concentric circles centered at the cell body and separated by 20 mm intervals were superimposed upon traces of apical and basilar dendrites; the number of dendritic intersections with each concentric circle was recorded.

### Immunoblotting

Brain extracts were prepared with ice-cold lysis buffer (0.25 M Tris [pH 7.5]) containing protease inhibitors (Protease Inhibitor Cocktail tablets, Roche Diagnostics, http://www.roche.com/div_diag.htm) and phosphatase inhibitors (Phosphatase Inhibitor Cocktails I & II, Sigma). Proteins were separated in 10% SDS-polyacrylamide gels and transferred onto nitrocellulose membranes. Blots were probed with antibodies to Akt (Cell Signaling, http://www.cellsignal.com), phospho-serine473-Akt (Cell Signaling), phospho-threonine308-Akt (Cell Signaling), and phospho-serine9-GSK3β (Cell Signaling) as well as those to GAPDH (Advanced ImmunoChemical, http://www.advimmuno.com) which was used as a loading control.

### Statistical Analysis

The Rotarod behavioral scores were subjected to statistical analysis using one-way or two-way ANOVA with repeated measures. For the Morris maze, escape latency was analyzed using two-way ANOVA with repeated measures. Probe trial was analyzed by one-way ANOVA followed by Student-Newman-Keuls post-hoc test to detect significant learning in each experimental group. One-way ANOVA was then performed to compare the search time for the training quadrant and multiple comparisons among all the groups were performed using Scheffe post hoc test. In the Pavlovian conditioned fear test, the percentage freezing was determined as described above, with a two-tailed t-test used to analyze the effects of treatment on percentage freezing. Sholl analysis data were analyzed using a multivariate analysis of variance (MANOVA). The MANOVA models considered both the effects of genotype and lithium treatment as well as the genotype and lithium treatment interaction effect. For a more direct analysis of the effect of lithium treatment, multivariate comparison of group means (control wild-type [Cont WT] versus lithium-treated wild-type [Li WT]; control knock-in [Cont KI] versus Cont WT; and Cont KI versus lithium-treated knock-in [Li KI]) was also performed using Hotelling T-statistic, utilizing the MANOVA fit to estimate the covariance. We performed t-tests (not assuming equal variances) and one-way repeated measures ANOVA using the StatView software (http://www.statview.com); R software (http://www.r-project.org) was utilized to perform the MANOVA. The other statistical analyses, including two-way repeated measures ANOVA, were performed with the SPSS statistical software package (http://www.spss.com).

## Results

### Effects of Lithium Carbonate Treatment on Motor Coordination in *Sca1^154Q/2Q^* Mice

**Figure 1:**
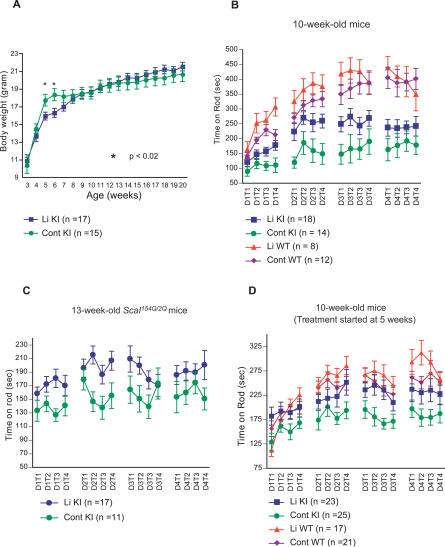
Lithium Treatment Improves Motor Coordination in *Sca1^154Q/2Q^* Mice (A) Body weights of *Sca1^154Q/2Q^* mice on a diet with (Li KI) or without (Cont KI) 0.2% lithium carbonate supplementation. *p < 0.02 by Student t-test. (B–D) Rotarod analysis. Ten-week-old naïve mice (B and D) and 13 week-old naïve *Sca1^154Q/2Q^* (C) mice were trained on the accelerating Rotarod in four trials per day (T1–T4) for 4 d (D1–D4). Lithium treatment was started either at the time of weaning (B and C) or at five weeks of age (D), when the *Sca1^154Q/2Q^* mice already show motor impairments. In both (B) and (D), repeated measures ANOVA revealed a significant time by day interaction for each of the four groups (unpublished data). Two-way repeated measures ANOVA indicated there were main effects of genotype (*p* < 0.001 for [B]; *p* = 0.002 for [D]) and treatment (*p* = 0.037 for [B] and *p* = 0.005 for [D]), but their interaction was not significant in (B) (*p* = 0.491) or (D) (*p* = 0.115). Cont KI, *Sca1^154Q/2Q^* mice on control diet; Li KI, lithium-treated *Sca1^154Q/2Q^* mice on lithium-supplemented diet; Li WT, wild-type mice on lithium-supplemented diet; Cont WT, wild-type mice on control diet. All data are shown as mean ± SEM.

At three weeks of age, upon weaning from their mothers, *Sca1^154Q/2Q^* mice and their wild-type littermates were maintained on regular rodent chow or on a diet containing 0.2% lithium carbonate. This treatment gave a plasma concentration above the minimal therapeutic concentration (0.6 mM) for treatment of bipolar disorder in both wild-type (0.83 mM, *n* = 1, at 15 wk; 0.87 mM, *n* = 1, at 24 wk) and *Sca1^154Q/2Q^* mice (0.91 ± 0.04 mM, *n* = 12, at 15 wk). Wild-type and *Sca1^154Q/2Q^* mice receiving lithium carbonate appeared to consume more water (possibly due to lithium's diuretic action) and gained less weight between three and six weeks of age than did wild-type and mutant mice on a control diet, respectively. After seven weeks of age, however, both treated groups gained more weight than their respective untreated counterpart until the body weight of treated and untreated groups became similar at ten weeks of age (Figure 1A).

In order to examine the effect of lithium treatment on motor coordination, we tested *Sca1^154Q/2Q^* mice of C57BL/6J-129/SvEv mixed background on the accelerating rotating rod. Because body weight significantly affects the performance of mice on the rotating rod, we performed this behavioral assay after ten weeks of age when the weights of lithium-treated mutant and control animals were comparable to their respective untreated counterparts. Two-way repeated measures ANOVA performed on the data set containing lithium-treated and untreated mutant animals revealed that there was a significant treatment effect (*p* = 0.0376), mediated by the enhanced performance of the lithium-treated mutant animals compared to mutant animals reared on a control diet at ten weeks of age (Figure 1B). In contrast, lithium did not produce a significant improvement in the performance of wild-type mice in comparison to wild-type mice on a control diet (*p* = 0.198, Figure 1B). To see if these findings were reproducible, we tested another batch of lithium-treated mutant animals whose genetic background was closer to C57BL/6J after five backcrosses (N5 generation). These animals showed significantly better performance on the accelerating Rotarod at 13 weeks of age than untreated age- and weight-matched mutant animals (*p* = 0.0192, by repeated measures ANOVA, Figure 1C). These findings were reproduced in a second cohort of backcrossed *Sca1* KI mice of N6 generation (unpublished data). These data suggest that continued lithium treatment improved motor coordination in the *Sca1^154Q/2Q^* mice regardless of the genetic background of the animals.

*Sca1^154Q/2Q^* mice developed motor incoordination as early as five weeks of age [18]. In order to examine if lithium is effective after onset of symptoms, we evaluated the performance of backcrossed (N8 or above) mutant animals that were given the lithium-containing chow after 5 weeks of age. These animals also showed significantly improved performance on the Rotarod at ten weeks of age compared to untreated age- and weight-matched mutant animals (*p* = 0.0358, by repeated measures ANOVA, Figure 1D). These results suggest that the lithium treatment is effective even when it is initiated postsymptomatically. More importantly, the reproducibility of the effects of lithium on four independent cohorts of *Sca1^154Q/2Q^* mice speaks to the positive effect of this drug on the coordination phenotype.

### The Performance of Lithium-Treated *Sca1^154Q/2Q^* Mice in Learning and Memory Tasks

**Figure 2:**
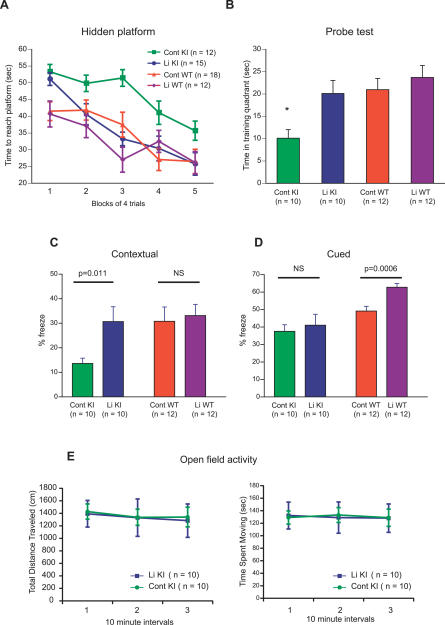
*Sca1^154Q/2Q^* Mice Reared on Lithium-Containing Chow Have Improved Learning and Memory Functions (A and B) Performance of nine- to 11-week old mice on the Morris hidden-platform water maze. *Sca1^154Q/2Q^* mice on the control diet (Cont KI) learned more slowly than those given lithium (Li KI). In a 60 s probe trial (B), Cont KI spent significantly less time in the training quadrant than Li KI (*p* = 0.012 by multiple comparisons using Scheffe's post hoc test), whereas Li KI spent similar amounts of time in the training quadrant compared to wild-type mice given lithium (Li WT) (*p* = 0.770) or wild-type mice on the control diet (Cont WT) (*p* = 0.847). (C and D) Pavlovian conditioned fear for *Sca1^154Q/2Q^* (KI) and wild-type (WT) mice treated with lithium (Li) or fed control chow (Cont). Percentage of time spent in freezing behavior during the contextual test (C) and the cued test (D) is shown. (E) Open-field activity for *Sca1^154Q/2Q^* mice. Locomotor activity of lithium-treated and untreated mutant mice was measured two weeks after the conditioned fear test. Each panel indicates total distance traveled (left graph) and time spent moving (right graph). All data are shown as mean ± SEM.

We next studied the effects of lithium treatment on cognitive function in 9- to 13-week-old *Sca1^154Q/2Q^* mice (Figure 2). We have previously shown that both spatial and Pavlovian learning and memory are impaired in *Sca1^154Q/2Q^* mice, as reflected by their performance on the Morris water maze and conditioned fear tests, respectively [18]. In the hidden platform version of the Morris water maze, nine- to 11-week-old lithium-treated *Sca1^154Q/2Q^* mice located the platform in significantly less time than those on a control diet (*p* < 0.0001, by repeated measures ANOVA, Figure 2A). During the probe trial on day 5, lithium-treated *Sca1^154Q/2Q^* mice spent more time in the training quadrant than the untreated mutants (Figure 2B). This difference was probably not attributable to increased motor activity, because lithium-treated *Sca1^154Q/2Q^* mice and untreated mutants displayed similar swim speeds during the probe trial (treated, 17.5 ± 0.8 cm/s; untreated, 16.2 ± 0.9 cm/s, mean ± SEM; *n* = 10 and 7 animals, respectively). The performance of wild-type mice was not significantly affected by lithium treatment. These results suggest that the lithium treatment improved spatial learning selectively in the *Sca1^154Q/2Q^* mice.

Two types of fear-conditioning paradigms, contextual and cued fear conditioning tests, were used to assay Pavlovian learning and memory in the lithium-treated *Sca1^154Q/2Q^* mice. These paradigms are thought to depend on plastic changes in the basolateral nucleus of the amygdala, with contextual fear conditioning also involving the hippocampus [19–21]. For the present study, the mice were placed in a chamber and twice given a mild foot shock paired with white noise. Twenty-four hours after this training, the mice were placed in the same chamber and their freezing behavior was monitored, in response to either the context (contextual fear conditioning) or the acoustic stimulus (white noise) delivered in a different context (cued fear conditioning). The degree of freezing indicates how well the animals remember the context or the cue. *Sca1^154Q/2Q^* mice on a control diet exhibited significant deficits in fear conditioning (low levels of freezing) in both the cued and contextual tests compared to wild-type mice (Figure 2C and 2D). Lithium-treated *Sca1^154Q/2Q^* mice showed significantly greater levels of freezing than the untreated mutant animals in the contextual test, whereas wild-type controls behaved similarly on the task independent of lithium treatment (Figure 2C). Lithium-treated and untreated *Sca1^154Q/2Q^* mice performed similarly in the cued test, but it is interesting that lithium-treated wild-type mice displayed increased freezing in the cued test (Figure 2D). The selective effect of the lithium treatment on the performance of *Sca1^154Q/2Q^* mice is not likely to be attributable to their decreased activity or the nonspecific sedative effects of the treatment, for two reasons. First, both lithium-treated and untreated wild-type mice showed similar levels of freezing in the context test. Second, both lithium-treated and untreated mutant animals showed similar levels of activity when they were tested in the open-field test (Figure 2E). Overall, these data indicate that lithium treatment selectively improved learning and memory measures in *Sca1^154Q/2Q^* mice. Specifically, the combined data from the Morris water maze and contextual fear conditioning support a positive effect of lithium on the hippocampus-dependent memory of *Sca1^154Q/2Q^* mice.

### Neuropathology of Lithium-Treated *Sca1^154Q/2Q^* Mice

**Figure 3:**
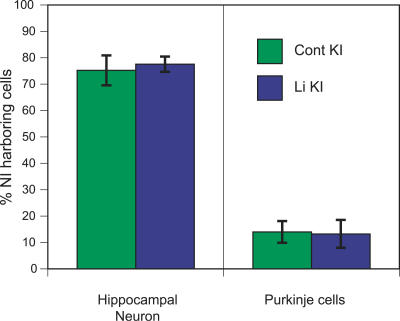
Lithium Treatment Does Not Affect NI Formation in *Sca1^154Q/2Q^* Mice The percentage of NI-harboring neurons is shown for 30- to 36-week-old mutant mice. Numbers of animals used for counting hippocampal neurons were six for lithium-treated *Sca1^154Q/2Q^* mice (Li KI) and four for *Sca1^154Q/2Q^* mice on the control diet (Cont KI); numbers of animals used for counting Purkinje cells were three for Li KI and three for Cont KI. Error bars indicate SEM.

Examination of hematoxylin-eosin-stained brain sections revealed no differences in gross morphology between lithium-treated and untreated *Sca1^154Q/2Q^* mice at 17–36 weeks of age. We additionally compared NI formation in the mutant brains, given that previous immunohistochemical studies with anti-ATXN1 antibodies demonstrated that *Sca1^154Q/2Q^* mice develop extensive NI formation in hippocampal neurons but delayed and less abundant NI formation in mutant Purkinje neurons [18]. In 30- to 36-week-old lithium-treated *Sca1^154Q/2Q^* mice, we found no significant difference in the percentage of hippocampal or Purkinje neurons harboring ATXN1-positive NI compared to littermate *Sca1^154Q/2Q^* mice on a control diet (by t-test, Figure 3), suggesting that the chronic lithium treatment did not significantly affect the magnitude or distribution of NI formation in the *Sca1^154Q/2Q^* mice.

To determine if lithium therapy has an effect on the neurodegenerative changes associated with SCA1 pathogenesis, we first analyzed dendritic arborization of the mutant Purkinje neurons quantitatively using anti-calbindin immunofluorescence [18]. Reduced dendritic arborization of Purkinje neurons was noted as early as nine weeks of age in the *Sca1^154Q/2Q^* mice [18]. Both lithium-treated and control mutant animals displayed similar intensities of calbindin immunofluorescence throughout the Purkinje cell layer and molecular layer of the cerebellum at 17 weeks of age, indicating that lithium treatment did not significantly affect dendritic degeneration of Purkinje cells in *Sca1^154Q/2Q^* mice (unpublished data).

**Figure 4:**
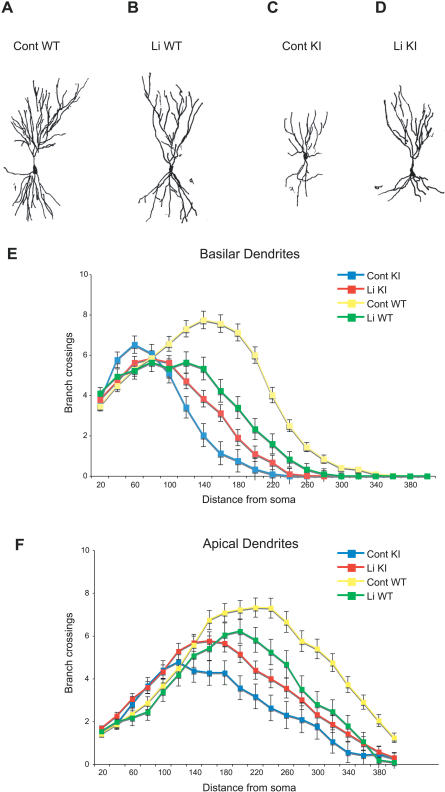
Lithium Treatment Partially Rescues Dendritic Pathology in Hippocampal CA3 Pyramidal Neurons of *Sca1^154Q/2Q^* Mice (A–D*)* Camera lucida drawings of representative pyramidal cell neurons from each experimental group. Shown are wild-type, control diet (Cont WT; *n* = 5 mice, 50 neurons) (A); wild-type, lithium diet (Li WT; *n* = 3 mice, 30 neurons) (B); *Sca1^154Q/2Q^* knock-in, control diet (Cont KI; *n* = 3, 30 neurons) (C); and (D) *Sca1^154Q/2Q^* knock-in, lithium diet (Li KI; *n* = 6, 60 neurons). (E and F) The effects of lithium treatment on the branching pattern of basilar (E) and apical (F) dendrites at increasing distances from the soma are shown. Genotype and lithium treatment significantly altered both basilar (E) and apical dendritic intersections (F) (genotype effect: *p* < 0.001, for basilar and apical; lithium treatment effect: *p* = 0.025 for basilar, *p* = 0.018 for apical). Sholl analysis additionally indicated a significant interaction between lithium treatment and genotype (*p* < 0.001 for basilar, *p* = 0.053 for apical). Values represent mean ± SEM.

*Sca1^154Q/2Q^* mice not only suffer from deficits in hippocampus-dependent learning and memory, but also show hippocampal atrophy in the absence of apparent cell loss [18]. In order to examine the dendritic morphology of the mutant hippocampal pyramidal neurons and determine whether differences in performance on hippocampus-dependent tasks correlated with improvements in cellular pathology, we examined dendritic arborization of CA3 pyramidal neurons by Sholl analysis of Golgi preparations [22]. Inspection of camera lucida drawings of pyramidal neurons from *Sca1^154Q/2Q^* or wild-type mice on control or lithium diet revealed differences in dendritic arborization (Figure 4A–4D). Quantitative assessment of Sholl data using MANOVA indicated that there were significant genotype and lithium treatment effects as well as significant interaction between genotype and lithium treatment for basilar, and to a lesser extent, apical dendrites (interaction effect: *p* < 0.001, basilar; *p* = 0.053, apical [Figure 4E and 4F]). Specifically, by multivariate contrast analysis, *Sca1^154Q/2Q^* mice had significantly fewer branch intersections than wild-type mice, and lithium treatment significantly improved this measure (Cont KI versus Cont WT: *p* < 0.001, basilar and apical; Cont KI versus Li KI, *p* = 0.007, basilar; *p* = 0.031, apical, Figure 4E and 4F). These results suggest that chronic lithium treatment, which was started from the time of weaning, partially rescued the degenerative changes seen in the hippocampal CA3 neurons of *Sca1^154Q/2Q^* mice. It is of note that although chronic lithium therapy decreased dendritic branching in wild-type animals (Cont WT versus Li WT: *p* < 0.001, basilar; *p* = 0.024, apical [Figure 4E and 4F]), it proved protective in the context of the SCA1 mutation.

### Levels of *Icmt/Pccmt* mRNA in Lithium-Treated *Sca1^154Q/2Q^* Mice

**Figure 5:**
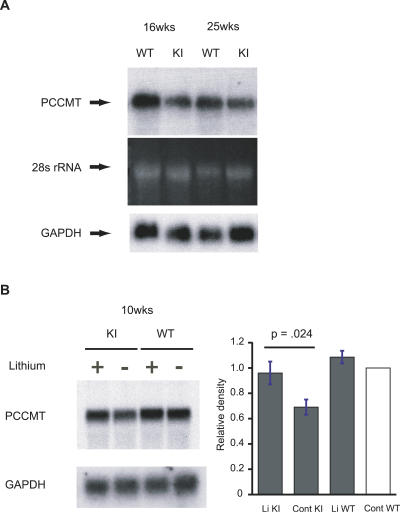
Lithium Treatment Increases *Icmt/Pccmt* mRNA Levels in *Sca1^154Q/2Q^* Cerebellum (A) Temporal profile of *Icmt/Pccmt* mRNA down-regulation in the *Sca1^154Q/2Q^* cerebellum. Cerebellar expression levels of *Icmt/Pccmt* mRNA in *Sca1^154Q/2Q^* mice and wild-type littermates were compared by Northern analysis. Ethidium bromide-stained RNA gel images are shown as a loading control, which was confirmed by hybridization with *Gapdh*. 25 μg of total RNA obtained from one cerebellum was loaded in each lane. Densitometric analysis using *Gapdh* as a control revealed a relative abundance of 0.465 for the 25 week-old *Sca1^154Q/2Q^* cerebellum compared to the respective wild-type littermate cerebella. (B) Northern blots of cerebellar RNA (25 μg) from two *Sca1^154Q/2Q^* mice and two wild-type littermates treated with lithium (+) or control diet (−) at ten weeks of age (blots on left) were probed with *Icmt/Pccmt* and *Gapdh* cDNAs. Quantitative analysis from four independent experiments (bar graph on right), in which total cerebellar RNAs were obtained from one or two *Sca1^154Q/2Q^* mice and one or two wild-type littermates for each experimental group (in total, six *Sca1^154Q/2Q^* mice on lithium diet, four *Sca1^154Q/2Q^* mice on control diet, six wild-type mice on lithium diet, and six wild-type mice on control diet), indicates *Icmt/Pccmt* expression was significantly increased in lithium-treated *Sca1^154Q/2Q^* mice (bar graph on right). Error bars indicate mean ± SEM.

Altered expression of specific genes has been implicated in the pathogenesis of several polyglutamine diseases [1,10]. In the case of SCA1, down-regulation of the *isoprenylcysteine carboxyl methyltransferase* (*Icmt;* alternatively, *Pccmt*) gene in Purkinje cells is one of the earliest known molecular alterations occurring in SCA1 transgenic mice [10]. In order to confirm that alterations in expression of *Icmt/Pccmt* occur in the *Sca1^154Q/2Q^* mice as well, we examined *Icmt/Pccmt* mRNA expression in the cerebellum of *Sca1^154Q/2Q^* and wild-type mice by Northern analysis (Figure 5). In *Sca1^154Q/2Q^* cerebellum, the expression of *Icmt/Pccmt* mRNA was down-regulated as early as ten weeks of age and markedly decreased by 16 weeks of age (Figure 5A and 5B). Because *Icmt/Pccmt* down-regulation is one of the earliest known alterations in both SCA1 transgenic mice and *Sca1^154Q/2Q^* mice, and because this gene is also down-regulated in human postmortem samples, we consider it a reliable early marker of transcriptional dysregulation and disease pathogenesis. Lithium treatment of the *Sca1^154Q/2Q^* mice resulted in almost normal levels of *Icmt/Pccmt* mRNA at ten weeks of age (Figure 5B). Densitometric analysis of the blots from four independent experiments revealed *Icmt/Pccmt* expression to be significantly elevated in lithium-treated knock-in mice (10–28 weeks of age) in comparison with the untreated knock-in littermates (*p* = 0.024 by *t-*test, Figure 5B, right column). In fact, the levels of *Icmt/Pccmt* mRNA in treated mutant animals were close to normal (98% of untreated wild-type animals), based on analysis of four independent pairs (ten *Sca1^154Q/2Q^* and 12 wild-type mice, total). Thus, chronic lithium treatment resulted in restoration of the levels of one of the earliest molecular changes in *Sca1^154Q/2Q^* mice—cerebellar transcriptional dysregulation of *Icmt/Pccmt* almost to normal.

### Effects of Lithium on GSK3β Phosphorylation and Akt Phosphorylation in *Sca1^154Q/2Q^* Mouse Brains

**Figure 6:**
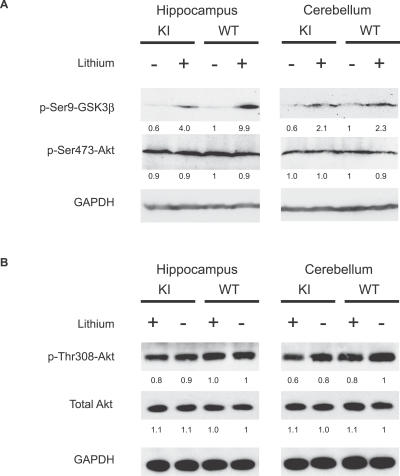
Phospho-Ser-9-GSK3β, but not Phospho-Akt, was Up-Regulated in Lithium-Treated Mouse Brains (A) Phospho-Ser-9-GSK3β, but not phospho-Ser-473-Akt, was up-regulated in lithium-treated mouse brains. Shown are blots of hippocampal (left) and cerebellar (right) extracts from *Sca1^154Q/2Q^* mice (KI) and wild-type littermates (WT), with (+) or without (−) lithium treatment, immunolabeled with phospho-Ser9-GSK3β, phospho-Ser473-Akt, and GAPDH antibodies. Each number indicates relative intensity of the band compared to the intensity of wild-type mice without the treatment. (B) Phospho-Thr-308-Akt was not up-regulated in the lithium treated mouse brains. Shown are blots of hippocampal (left) and cerebellar (right) extracts from *Sca1^154Q/2Q^* mice (KI) and wild-type littermates (WT), with (+) or without (−) lithium treatment, immunolabeled with phospho-Thr308-Akt, total Akt, and GAPDH antibodies. Similar results were obtained from the extracts from the rest of the brains. Each number indicates relative intensity of the band compared to the intensity of wild-type mice without the treatment. On each immunoblot, individual lanes were loaded with a protein extract prepared from one animal and similar results were reproduced with one to three more independent experiments (two to four animals in total were used).

The above results suggest that one of the ways lithium treatment ameliorated the behavioral deficits and hippocampal neuropathology of *Sca1^154Q/2Q^* mice might be by affecting neuronal gene expression. There are two major pathways by which lithium is known to affect the activities of multiple transcription factors that control gene expression, and both of these pathways involve inhibitory effects on GSK3: lithium can act as a competitive inhibitor of Mg^2+^, thereby reducing GSK3 activity [14]; lithium also inhibits GSK3 by increasing the inhibitory phosphorylation of a Ser-9 residue in GSK3β or Ser-21 on GSK3α [14]. Chronic administration of lithium to mice has been shown to cause an increase in the phosphorylation of Ser-9 of GSK3β in vivo. We therefore examined the effects of lithium treatment on the phosphorylation of Ser-9 of GSK3β using hippocampal extracts from nine-week-old animals. Phospho-Ser-9-GSK3β levels were indeed greater in the hippocampi of lithium-treated mice (both wild-type and *Sca1^154Q/2Q^*) than in their untreated littermates (Figure 6A). Similar results were obtained from cerebellar extracts of these animals (Figure 6A).

The mechanism by which lithium enhances phospho-Ser-9-GSK3β levels has not been fully resolved. GSK3β is known to be a substrate of serine/threonine kinase Akt, and Akt is mostly regulated through the phosphoinositol-dependent phosphorylation of both its Ser-473 and Thr-308 residues, resulting in its activation. It has been reported that lithium might increase phosphorylation of either of the two residues through activation of phosphatidylinositol 3-kinase [24,25]. Other reports have postulated that GSK3β phosphorylation is increased through inhibition of a protein phosphatase that normally removes a phosphate from the regulatory serine [26]. To determine which of these two scenarios might account for enhanced levels of phospho-Ser-9-GSK3β, we examined phosphorylation of Akt on Ser-473 and Thr-308. We did not find enhanced levels of phospho-Ser-473-Akt (Figure 6A) nor phospho-Thr-308-Akt (Figure 6B) in the brains of lithium-treated mice (either *Sca1^154Q/2Q^* or wild-type).

## Discussion

We have shown that lithium carbonate mitigates the severe motor dysfunction and cognitive impairment observed in *Sca1^154Q/2Q^* mice. Motor dysfunction improved even when treatment was initiated after the onset of symptoms. Sholl analysis revealed dendritic pathology in hippocampal CA3 pyramidal neurons of *Sca1^154Q/2Q^* mice, which was partially rescued upon lithium treatment. In addition, we have shown that the expression of the *Icmt/Pccmt* gene, whose down-regulation is an early marker of SCA1 pathology, is elevated after lithium treatment in *Sca1^154Q/2Q^* mice. The finding of increased phospho-GSK3β supports the hypothesis that these effects could be mediated at least in part through transcription.

Lithium has been reported to exert its neuroprotective effects predominantly by inhibiting apoptosis through inhibition of GSK3β [27]. Given the lack of evidence linking apoptosis with the pathogenesis of SCA1 [18,28,29], we think it is unlikely that the beneficial effects of lithium on neurological function arose from such antiapoptotic activities in *Sca1^154Q/2Q^* mice. There are, however, several other possible modes of activity by which lithium may improve the course of the disease in *Sca1^154Q/2Q^* mice. Lithium has diverse molecular targets (including GSK3β) that affect gene expression, and chronic lithium treatment has been reported to increase the DNA binding activity of the transcription factor AP-1 in rat brains [30]. Since alteration in gene expression is an early step in SCA1 pathogenesis, it is conceivable that lithium could exert some of its neuroprotective effect by favorably affecting gene expression in the mutant mice. Although it is unclear whether the enhanced levels of *Icmt/Pccmt* mRNA contributed to the improved behavioral profile of treated *Sca1^154Q/2Q^* mice, the normalization of its levels suggest that lithium might help also correct some of the transcriptional dysregulation underlying SCA1.

Our Sholl analysis data suggest that the lithium treatment could improve cognitive dysfunction in *Sca1^154Q/2Q^* mice, possibly by partially rescuing dendritic atrophy of the mutants' hippocampal pyramidal neurons. It is interesting that chronic lithium treatment has been reported to prevent the stress-induced decrease in dendritic length in CA3 hippocampal neurons [31]. Thus, it is plausible that the pathogenic mechanism of mutant ATXN1-induced dendritic atrophy shares common downstream pathways with that of stress-induced dendritic remodeling*,* which was shown to depend on excitatory amino acid activity [32]. Lithium could also affect neurotransmission or neurogenesis. Chronic lithium treatment up-regulates synaptosomal uptake of glutamate [33], and lithium treatment enhances long-term potentiation in the rat dentate gyrus [34]. It has also been reported that chronic administration of lithium enhances neurogenesis in the dentate gyrus of adult rodents [35].

Our trials in a faithful mouse model of SCA1, which expresses endogenous levels of mutant, full-length ATXN1 protein in a proper spatial and temporal pattern and reproduces many features of the human disease, strongly suggest that the therapeutic potential of lithium in this disease deserves serious consideration. Importantly, the lithium trial improved the motor impairment even when it was started after the onset of disease in *Sca1^154Q/2Q^* mice. However, it remains unclear whether all the beneficial effects of lithium would last throughout the disease course. When we tested the mice on the rotarod at 20 weeks of age, we found signs of improved performance but the differences failed to reach statistical significance between the treated and untreated mutant mice (*p* = 0.102, by repeated measures ANOVA). Thus, lithium may not confer long-lasting benefits. Nonetheless, we cannot rule out whether using a less challenging paradigm might be required to delineate differences in the motor performance at this stage. We did not observe a significant improvement in life span (unpublished data), but life span measurement could also be confounded by various factors. For instance, it has been shown that long-term lithium treatment could disturb serum ionic balance in rodents [36] such that effects on normal functions of organs outside the central nervous system could have confounded life span measures. Further, because the *Sca1^154Q/2Q^* mice express a CAG tract that is much longer than typically seen in human patients (in order to produce a phenotype during the short life span of the mouse [37]), their disease might be more aggressive than the human condition. Finally, the therapeutic and toxic potential of lithium have been well documented; tremor and lack of coordination are among the side effects reported, particularly when the dose is not carefully monitored [38]. Because such side effects could be of concern when treating patients with a disorder of cerebellar dysfunction, we evaluated tremor events in mice using a tremor monitoring system. We did not find any indication of increased tremor in chronically lithium-treated *Sca1^154Q/2Q^* or wild-type mice (unpublished data), suggesting that the lithium trial at the doses used in our study did not induce tremors even in the context of an *ATXN1* mutation.

It is interesting to note that lithium has produced beneficial effects in a cellular, *Drosophila,* and mouse model of Huntington disease toxicity in which fragments of polyglutamine expanded mutant huntingtin were expressed. Lithium chloride treatment reduced toxicity induced by overexpression of Huntington disease exon 1 fragment with 74 glutamines in neuronal and non-neuronal cell lines [11], and attenuated toxicity of the N-terminal part of mutant huntingtin with 120 glutamine repeats in *Drosophila* [39]. Wood and Morton recently showed that chronic lithium treatment improved motor function in a transgenic mouse model of Huntington disease (the R6/2 line) that expresses exon 1 of the human *huntingtin* gene with approximately 150 CAG repeats [40]. In this study, lithium improved the performance of the mice in one behavioral paradigm, the rotating rod, but only in animals treated postsymptomatically [40]. It remains to be seen if lithium therapy would be beneficial in mouse models of HD that express the full-length protein. This experiment is critical because protein context has increasingly been shown to be important in the pathogenesis of polyglutamine disorders, such that normal function of the full-length host protein may determine molecular pathways, pathogenic progression, and potential therapeutic targets [41,42]. Here, we show the beneficial effects of chronic lithium treatment on multiple measures in an SCA1 disease model—*Sca1^154Q/2Q^* mice—that express the full-length mutant protein in the endogenous spatiotemporal pattern and reproduce most features of human SCA1. Future studies using other polyglutamine diseases, particularly the knock-in mouse models, will be necessary to determine whether lithium will prove effective for polyglutamine diseases in general or will have a more limited therapeutic role.

Lithium's amelioration of the phenotypes of *Sca1^154Q/2Q^* mice may occur through more than one mechanism. Consistent with this idea is the finding that several independent neurological functions are improved in lithium-treated *Sca1^154Q/2Q^* mice along with an apparent differential morphological effect on Purkinje cells and hippocampal pyramidal neurons. Despite significant advances in understanding the molecular pathogenesis of SCA1, effective treatments have not yet been revealed. The present study suggests that lithium might safely improve the motor coordination and cognitive function of SCA1 patients, perhaps providing them with better quality of life. Translating successful drug trials in mouse models into a clinical context is not always an easy task [43], but the fact that lithium has been efficacious in treating other human diseases raises hope that it may be useful for therapeutic trials in SCA1 patients, irrespective of its mechanism of action.

## Supporting Information

Alternative Language Abstract S1 Translation into French by Celine Coutte (22 KB DOC)

Alternative Language Abstract S2 Translation into Japanese by Kei Watase (142 KB PDF)

Alternative Language Abstract S3 Translation into Spanish by Roula Zoghbi (21 KB DOC)

## Abbreviations

CS: conditioned stimulus
MANOVA: multivariate analysis of variance
NI: neuronal intranuclear inclusion
SCA1: spinocerebellar ataxia type 1
US: unconditioned stimulus

## References

[pmed-0040182-b001] Gatchel JR, Zoghbi HY (2005). Diseases of unstable repeat expansion: Mechanisms and common principles. Nat Rev Genet.

[pmed-0040182-b002] Zoghbi HY, Orr HT (1995). Spinocerebellar ataxia type1. Semin Cell Biol.

[pmed-0040182-b003] Kish SJ, el-Awar M, Schut L, Leach L, Oscar-Berman M (1988). Cognitive deficits in olivopontocerebellar atrophy: Implication for the cholinergic hypothesis of Alzheimer's dementia. Ann Neurol.

[pmed-0040182-b004] Bürk K, Globas C, Bösch S, Klockgether T, Zühlke C (2003). Cognitive deficits in spinocerebellar ataxia type 1, 2, and 3. J. Neurol.

[pmed-0040182-b005] Orr HT, Zoghbi HY (2001). SCA1 molecular genetics: A history of a 13 year collaboration against glutamines. Hum Mol Genet.

[pmed-0040182-b006] Klement IA, Skinner PJ, Kaytor MD, Yi H, Hersch SM (1998). Ataxin-1 nuclear localization and aggregation: Role in polyglutamine-induced disease in SCA1 transgenic mice. Cell.

[pmed-0040182-b007] Emamian ES, Kaytor MD, Duvick LA, Zu T, Tousey SK (2003). Serine 776 of ataxin-1 is critical for polyglutamine-induced disease in *SCA1* transgenic mice. Neuron.

[pmed-0040182-b008] Chen H–K, Fernandez-Funez P, Acevedo SF, Lam YC, Kaytor MD (2003). Interaction of Akt-phosphorylated ataxin-1with 14-3-3 mediates neurodegeneration in spinocerebellar ataxia type 1. Cell.

[pmed-0040182-b009] Cummings CJ, Sun Y, Opal P, Antalffy B, Mestril R (2001). Overexpression of inducible Hsp70 chaperone suppresses neuropathology and improves motor function in SCA1 mice. Hum Mol Genet.

[pmed-0040182-b010] Lin X, Antalffy B, Kang D, Orr HT, Zoghbi HY (2001). Polyglutamine expansion downregulates specific neuronal genes before pathologic changes in SCA1. Nat Neurosci.

[pmed-0040182-b011] Carmichael J, Sugars KI, Bao YP, Rubinsztein DC (2002). Glycogen synthase kinase-3beta inhibitors prevent cellular polyglutamine toxicity caused by the Huntington's disease mutation. J Biol Chem.

[pmed-0040182-b012] Chen G, Zeng W-Z, Yuan P-X, Huang L-D, Jiang Y-M (1999). The mood-stabilizing agents lithium and valproate robustly increase the levels of the neuroprotective protein bcl-2 in the CNS. J Neurochem.

[pmed-0040182-b013] Pilcher HR (2003). Drug research: The ups and downs of lithium. Nature.

[pmed-0040182-b014] Jope RS (2003). Lithium and GSK-3: One inhibitor, two inhibitory actions, multiple outcomes. Trend Pharmacol Sci.

[pmed-0040182-b015] Chen G, Masana MI, Manji HK (2000). Lithium regulates PKC-mediated intracellular cross-talk and gene expression in the CNS in vivo. Bipolar Disord.

[pmed-0040182-b016] Phiel CJ, Wilson CA, Lee VM-Y, Klein PS (2003). GSK3α regulates production of Alzheimer's disease amyloid-β peptide. Nature.

[pmed-0040182-b017] Chuang D-M, Chen R-W, Chalecka-Franaszek E, Ren M, Hashimoto R (2002). Neuroprotective effects of lithium in cultured cells and animal models of diseases. Bipolar Disord.

[pmed-0040182-b018] Watase K, Weeber EJ, Xu B, Antalffy B, Yuva-Paylor L (2002). A long CAG repeat in the mouse *Sca1* locus replicates SCA1 features and reveals the impact of protein solubility on selective neurodegeneration. Neuron.

[pmed-0040182-b019] Bordi F, Marcon C, Chiamulera C, Reggiani A (1996). Effects of the metabotropic glutamate receptor antagonist MCPG on spatial and context-specific learning. Neuropharmacology.

[pmed-0040182-b020] Favata MF, Horiuchi KY, Manos EJ, Daulerio EJ, Stradley DA (1998). Identification of a novel inhibitor of mitogen-activated protein kinase kinase. J Biol Chem.

[pmed-0040182-b021] Hargreaves EL, Cain DP (1992). Hyperactivity, hyper-reactivity, and sensorimotor deficits induced by low doses of the N-methyl-D-aspartate non-competitive channel blocker MK801. Behav Brain Res.

[pmed-0040182-b022] Sholl DA (1955). The organization of the visual cortex in the cat. J Anat.

[pmed-0040182-b023] Luthi-Carter R, Strand A, Peters NL, Solano MS, Hollingsworth ZR (2000). Decreased expression of striatal signaling genes in a mouse model of Huntington's disease. Hum Mol Genet.

[pmed-0040182-b024] Chalecka-Franaszek E, Chuang DM (1999). Lithium activates the serine/threonine kinase Akt-1 and suppresses glutamate-induced inhibition of Akt-1 activity in neurons. Proc Natl Acad Sci U S A.

[pmed-0040182-b025] Beaulieu J-M, Sotnikova TD, Yao W-D, Kockeritz L, Woodgett JR (2004). Lithium antagonizes dopamine-dependent behaviors mediated by an AKT/glycogen synthase kinase 3 signaling cascade. Proc Natl Acad Sci U S A.

[pmed-0040182-b026] Song L, De Sarno P, Jope RS (2002). Central role of glycogen synthase kinase-3β in endoplasmic reticulum stress-induced caspase-3 activation. J Biol Chem.

[pmed-0040182-b027] Li X, Bijur GN, Jope RS (2002). Glycogen synthase kinase-3β, mood stabilizers and neuroprotection. Bipolar Disord.

[pmed-0040182-b028] Shahbazian MD, Orr HT, Zoghbi HY (2001). Reduction of Purkinje cell pathology in SCA1 transgenic mice by p53 deletion. Neurobiol Dis.

[pmed-0040182-b029] Zoghbi HY, Orr HT, Scriver CR, Beaudet AL, Sly WS (2001). Spinocereballar ataxias. The metabolic and molecular bases of inherited disease.

[pmed-0040182-b030] Yuan PX, Chen G, Manji HH (1999). Lithium activates the c-Jun NH_2_-terminal kinases (JNKs) in vitro and in the CNS in vivo. J Neurochem.

[pmed-0040182-b031] Wood GE, Young LT, Reagan LP, Chen B, McEwen BS (2004). Stress-induced structural remodeling in hippocampus: Prevention by lithium treatment. Proc Natl Acad Sci U S A.

[pmed-0040182-b032] Magarinos AM, McEwen BS, Flugge G, Fuch E (1996). Chronic psychological stress causes apical dendritic atrophy of hippocampal CA3 pyramidal neurons in subordinate tree shrews. J Neurosci.

[pmed-0040182-b033] Dixon JF, Hokin LE (1998). Lithium acutely inhibits and chronically up-regulates and stabilizes glutamate uptake by presynaptic nerve endings in mouse cerebral cortex. Proc Natl Acad Sci U S A.

[pmed-0040182-b034] Son H, Yu IT, Hwang S-J, Kim JS, Lee S-H (2003). Lithium enhances long-term potentiation independently of hippocampal neurogenesis in the rat dentate gyrus. J Neurochem.

[pmed-0040182-b035] Chen G, Rajkowska G, Du F, Seraji-Bozorgzad N, Manji HK (2000). Enhancement of hippocampal neurogenesis by lithium. J Neurochem.

[pmed-0040182-b036] Chaudhuri-Sengupta S, Sarkar R, Maiti BR (2003). Lithium action on adrenomedullary and adrenocortical functions and serum ionic balance in different age groups of albino rats. Arch Physiol Biochem.

[pmed-0040182-b037] Lorenzetti D, Watase K, Xu B, Matzuk MM, Orr HT (2000). Repeat instability and motor incoordination in mice with a targeted expanded CAG repeat in the Sca1 locus. Hum Mol Genet.

[pmed-0040182-b038] Freeman MP, Freeman SA (2006). Lithium: Clinical considerations in internal medicine. Am J Med.

[pmed-0040182-b039] Berger Z, Ttofi EK, Michel CH, Pasco MY, Tenant S (2005). Lithium rescues toxicity of aggregate-prone proteins in *Drosophila* by perturbing Wnt pathway. Hum Mol Genet.

[pmed-0040182-b040] Wood NI, Morton AJ (2003). Chronic lithium chloride treatment has variable effects on motor behavior and survival of mice transgenic for the Huntington's disease mutation. Brain Res Bull.

[pmed-0040182-b041] Slow EJ, Graham RK, Osmand AP, Devon RS, Lu G (2005). Absence of behavioral abnormalities and neurodegeneration in vivo despite widespread neuronal huntingtin inclusions. Proc Natl Acad Sci U S A.

[pmed-0040182-b042] Graham RK, Deng Y, Slow EJ, Haigh B, Bissada N (2006). Cleavage at the caspase-6 site is required for neuronal dysfunction and degeneration due to mutant Huntingtin. Cell.

[pmed-0040182-b043] Watase K, Zoghbi HY (2003). Modelling brain diseases in mice: The challenge of design and analysis. Nat Rev Genet.

