# Supplementary material for: Lithium Therapy Improves Neurological Function and Hippocampal Dendritic Arborization in a Spinocerebellar Ataxia Type 1 Mouse Model
Source: PLoS Med. 2007 May 29;4(5):e182. doi: 10.1371/journal.pmed.0040182 (PMC1880853; doi:10.1371/journal.pmed.0040182)
Supplement: Alternative Language Abstract S1 — (22 KB DOC) [file pmed.0040182.sd001.doc]

Résumé

Contexte :

L’ataxie spinocérébelleuse autosomique dominante de type 1 (SCA1) est une maladie génétique neurodégénerative caractérisée par une détérioration progressive des fonctions motrices et cognitives. L’expression de la protéine ATAXIN1 présentant une extension polyglutaminique cause la maladie et la pathogenèse de SCA1 -bien que multifactorielle- a très certainement pour origine un mauvais repliement de l’ATAXIN1. Ce mauvais repliement aurait des conséquences directes sur la fonction de la protéine et ses interactions avec d’autres partenaires, et mènerait à des dérèglements transcriptionnels de l’ADN. Etant donné qu’il a été montré que le lithium exerce des effets neuroprotecteurs dans un certain nombre de cas et ce, en affectant l’expression des gènes, nous avons testé l’efficacité d’un traitement au lithium sur un model de souris knock-in pour SCA1 (souris *Sca1^154Q/2Q^*), model qui reproduit beaucoup de caractéristiques de la maladie chez l’homme.

Méthodologies et observations :

Des souris *Sca1^154Q/2Q^* ainsi que des souris de phénotype sauvage et ont été nourries avec des croquettes classiques ou avec des croquettes contenant 0.2% de carbonate de lithium. Le régime supplémenté en carbonate de lithium a montré une amélioration de la coordination motrice, de l’apprentissage, et de la mémoire chez les souris *Sca1^154Q/2Q^*. De plus, les améliorations motrices ont été observées lorsque le traitement a été initié aussi bien avant qu’après apparition des symptômes. Sur le plan neuropathologique, le traitement au lithium atténue la réduction des branchements dendritiques des neurones pyramidaux de l’hippocampe habituellement observée chez les souris mutantes. Nous avons aussi pu constater que le traitement au lithium restaure les niveaux de *Pccmt*, dont la régulation négative est un marqueur précoce de la toxicité des mutants ATAXIN1.

Conclusion :

L’effet du lithium sur un marqueur habituellement altéré de façon précoce dans l’évolution de la pathogénie de SCA1, ainsi que ses effets positifs sur différents critères du comportement et sur les neuropathologies de l’hippocampe dans un model animal valide de la maladie SCA1 font du lithium un excellent candidat pour le traitement des patients atteints de la maladie.
