# Supplementary material for: Lithium Therapy Improves Neurological Function and Hippocampal Dendritic Arborization in a Spinocerebellar Ataxia Type 1 Mouse Model
Source: PLoS Med. 2007 May 29;4(5):e182. doi: 10.1371/journal.pmed.0040182 (PMC1880853; doi:10.1371/journal.pmed.0040182)
Supplement: Alternative Language Abstract S2 — (142 KB PDF) [file pmed.0040182.sd002.pdf]

概要

背景

脊髄小脳変性症１型(SCA1)は進行性の運動及び認知機能障害を主徴とする優性遺伝性神経変性疾患である。SCA1 はアタキシン１に存在するポリグルタミンの伸長によってひき起こされるが、その病態発症機構はアタキシン１のミスフォールディングによるタンパク間相互作用の機能的変化に端を発する複雑な過程を経るものと考えられ、結果として、遺伝子発現調節に異常をきたす。リチウムはさまざまな病態において、おそらく遺伝子発現に影響を及ぼすことにより、神経保護作用を示すことが示されてきたので、我々は SCA1 の病態の多くの特徴をマウスで再現するノックインマウスモデル($Sca1^{154Q/2Q}$ マウス)を用いてリチウムによる治療の有効性を検討した。

方法と結果

$Sca1^{154Q/2Q}$ マウス及び同腹の野生型マウスに 0.2%炭酸リチウムを含有する食餌または通常の食餌を与えた。食餌への炭酸リチウムの添加により、$Sca1^{154Q/2Q}$ マウスの協調運動障害、学習記憶障害が改善した。重要なことに、リチウム投与を症状の発現以前に開始しても、症状の発現後に開始しても協調運動障害に対する効果が認められた。病理学的には、海馬錐体細胞の樹状突起の分枝の減少がリチウム投与により改善していた。さらに、リチウムの投与は変異アタキシン１による神経毒性の初期の指標として知られている *Pccmt* 遺伝子の発現の低下を抑制する。

結論

SCA1 の病態を再現するモデルマウスにおいて、リチウムの投与が行動学的異常や海馬の神経病理所見を改善し、病態の初期の指標の改善にも有効であったことから、リチウムは SCA1 患者に対しても有効な候補薬剤となりうることが示された。
