# Supplementary material for: Lithium Therapy Improves Neurological Function and Hippocampal Dendritic Arborization in a Spinocerebellar Ataxia Type 1 Mouse Model
Source: PLoS Med. 2007 May 29;4(5):e182. doi: 10.1371/journal.pmed.0040182 (PMC1880853; doi:10.1371/journal.pmed.0040182)
Supplement: Alternative Language Abstract S3 — (21 KB DOC) [file pmed.0040182.sd003.doc]

**Ataxia Espinocerebelar Tipo I (SCA1) es una enfermedad neurológica degenerativa con herencia autosómica dominante, caracterizada por disfunción cognitiva y discapacidad motora progresiva. SCA1 es causada por la expansión de un tracto de poliglutaminas en ATAXIN1, y su patogénesis conlleva un proceso multifactorial que probablemente es iniciado por cambios en la conformación estructural nativa de ATAXIN1, con consecuencias funcionales en sus interacciones proteicas, culminando en desregulación transcripcional. Como se ha demostrado previamente que litio ejerce efectos neuroprotectores bajo una variedad de condiciones, posiblemente afectando la expresión de genes, examinamos la eficacia del tratamiento de litio en un modelo de ratón “knock-in” de SCA1 (ratones *Sca1^154Q/2Q^*) que replica muchas de las características de la enfermedad humana.**

**Métodos y Hallazgos**

**Los ratones *Sca1^154Q/2Q^*y sus compañeros de camada fueron alimentados con comida para ratones regular o comida que contiene 0.2 % carbonato de litio.  El suplemento alimenticio con carbonato de litio resultó en una mejoría en coordinación motora, y en aprendizaje y memoria de los ratones *Sca1^154Q/2Q^*.  En gran medida, esta mejoría motora fue vista tanto cuando el tratamiento fue iniciado presintomáticamente, como después del comienzo de los síntomas.  Neuropatológicamente, el tratamiento con litio atenuó la reducción de las ramas dendríticas de las neuronas piramidales en el hipocampo de los ratones mutantes. Además, informamos que el tratamiento con litio restaura los niveles de *Pccmt*, cuya bajo-regulación es uno de los primeros indicadores de neurotoxicidad causada por ATAXIN1 mutante.**

**Conclusión**

**Los efectos de litio en la expresión del indicador Pccmt durante el curso de la patogénesis de SCA1, en combinación con su efecto positivo en múltiples medidas de comportamiento y en la neuropatología hipocampal de un modelo animal auténtico de la enfermedad, lo hacen un candidato excelente para un potencial tratamiento terapéutico en pacientes humanos de SCA1.**
